# Vitamin D deficiency and metabolic syndrome in elderly Chinese individuals: evidence from CLHLS

**DOI:** 10.1186/s12986-020-00479-3

**Published:** 2020-07-29

**Authors:** Ling Liu, Zhaojin Cao, Feng Lu, Yingchun Liu, Yuebin Lv, Yingli Qu, Heng Gu, Chengcheng Li, Jiayi Cai, Saisai Ji, Yawei Li, Feng Zhao, Xiaoming Shi

**Affiliations:** 1 National Institute of Environmental Health, Chinese Center for Disease Control and Prevention, #29 Nanwei Road, Xicheng, Beijing, 100050 China; 2 Beijing Municipal Health Commission Information Center, (Beijing Municipal Health Commission Policy Research Center), Beijing, 100034 China

**Keywords:** Chinese elderly people, Vitamin D, Metabolic syndrome, Elevated waist circumference

## Abstract

**Objectives:**

Both low vitamin D status and metabolic syndrome (MetS) are worldwide concerns, and low 25-hydroxyvitamin D [25(OH)D] levels are associated with MetS; however, related epidemiological evidence based on elderly Chinese individuals, especially those over 80 years of age, is limited. In the present study, we aimed to evaluate the association between serum 25(OH)D and MetS in elderly Chinese individuals.

**Method:**

Serum 25(OH)D was measured in a cross-sectional sample of 2493 elderly people aged 65–112 years from eight areas of China in which the density of centenarians is exceptionally high. MetS was diagnosed according to blood pressure, lipid, and blood sugar levels; waist circumference; and body mass index (BMI). Adjusted multivariable logistic regression was used to analyze the associations between vitamin D and MetS based on different diagnostic criterias.

**Results:**

A total of 890 (35.7%) of the recruited elderly individuals had insufficient levels of vitamin D, and 1029 participants (41.3%) were vitamin D deficient. High serum vitamin D concentrations were associated with a low prevalence of MetS according to the modified Adult Treatment Panel III (ATP III) criteria for adequate versus deficient vitamin D levels (OR: 0.63, 95% CI: 0.45, 0.88) and inadequate versus deficient vitamin D levels (OR: 0.70, 95% CI: 0.52, 0.92). Each 10 ng/ml increase in serum vitamin D was significantly associated with a decreased prevalence of MetS according to the modified ATP III criteria for people with normal waist circumference (WC) (OR: 0.55, 95% CI: 0.43,0.71). However, no significant statistical correlation was found among elderly people with a high WC. Additionally, in the analysis of the individual components, the ORs of adequate versus deficient vitamin D levels were 0.46 (95% CI: 0.30, 0.71) for elevated triglycerides and 0.64 (95% CI: 0.42, 0.97) for reduced high-density lipoprotein cholesterol (HDL-C) after adjustment for other components.

**Conclusions:**

Vitamin D deficiency is very common among elderly Chinese individuals. Vitamin D deficiency may be a risk factor for MetS; however, the association was only statistically significant among elderly people with noncentral obesity. Further studies are needed to examine the causal direction of the association.

## Introduction

As a fat-soluble vitamin, vitamin D is traditionally thought to regulate the metabolism of calcium and phosphorus in the body. However, emerging data suggest that vitamin D levels are also closely associated with many nonskeletal diseases, including cancer, infection, cardiovascular disease and metabolic syndrome (MetS) [1–4]. Vitamin D deficiency, which is a highly prevalent condition, has become a worldwide concern. Several large epidemiological studies have shown that vitamin D deficiency occurs in approximately 30 to 50% of the general population [5]. The prevalence of vitamin D deficiency among elderly Chinese individuals is even higher than that in other countries, at 79.7% in women and 64.0% in men [6]. With the increase of life expectancy per capita and the aggravation of global aging, he health conditions of and preventive health care for the elderly population are becoming increasingly important. Therefore, it is important to explore the health effects of vitamin D deficiency on elderly Chinese individuals.

MetS is a group of clinical syndrome characterized by insulin resistance, diabetes or impaired glucose regulation, obesity, abnormal lipid metabolism, hypertension and other metabolic diseases. The prevalence of MetS among elderly Chinese individuals increased from 50.4% in 2001 to 58.1% in 2010 [7]. Studying the risk factors for MetS, which is usually recognized as an important predictor of cardiovascular and cerebrovascular diseases and diabetes mellitus, is of great significance for improving the health condition of the population, especially that of the elderly population.

Several epidemiological studies have found that serum vitamin D levels are inversely associated with MetS in both Western and Asian populations [8–12]. Animal models and in vitro studies have revealed some relevant mechanisms of action of vitamin D on metabolic syndrome [13]. However, the results of epidemiological studies on the associations between serum vitamin D deficiency and MetS are inconsistent. In addition, many of the studies included only people younger than 60 years, and few of them focused on elderly individuals, especially people older than 80 years, which is the group with the highest prevalence of vitamin D deficiency and MetS [10, 14]. Because the study population of the only study focusing on an elderly Chinese population was selected from one or two of the most developed cities in China [9, 12], it is difficult for the results to be generalized to elderly individuals in other parts of China. Our study aimed to examine the relationship between vitamin D and MetS in eight longevity regions of eight provinces in China.

## Methods

### Study population

Data were based on the Chinese Longitudinal Healthy Longevity Survey (CLHLS), a community-based study designed to assess the health status and its influencing factors in elderly populations in longevity regions. In 2017, a biomarker substudy of the 2017–2018 CLHLS was conducted in eight longevity regions, including Xiayi County of Henan Province, Zhongxiang City of Hubei Province, Laizhou City of Shandong Province, Yongfu County of Guangxi Autonomous Area, Sanshui District of Guangdong Province, Mayang County of Hunan Province, Chengmai County of Hainan Province and Rudong County of Jiangsu Province. These eight areas are distributed across northern and southern China. Individuals aged 65 years and older (*N* = 2493) were recruited, and blood samples were obtained. The study was approved by the biomedical ethics committee of Peking University (IRB00001052–24713074). All participants signed written informed consent forms.

### Data collection

We collected information using a standardized questionnaire administered during an in-home interview. The information included demographic data such as age, gender, nationality, residential region, education level, marital status and health-related lifestyle information, such as current smoking, current alcohol consumption, physically active and medical conditions including self-reported diabetes and self-reported hypertension. We also recorded the sampling season for each participant. After the in-home interview, all participants were asked to undergo anthropometric measurements, which included height, weight, waist circumference (WC), hip circumference and two blood pressure measurements with at least a one-minute interval between them. Waist measurements were taken early in the morning on an empty stomach. The participants were asked to stand upright and relax his abdomen with gently breath. After having rested for 5 min under supervision of trained research assistants, arterial blood pressure was measured with mercury sphygmomanometer on the right arm at heart level of the seated subject. We would measure WC and SBP and DBP twice and take the mean value of each index. According to the criteria for Chinese individuals, we categorized body mass index (BMI) into two groups [15]: underweight and normal weight (< 24.0 kg/m^2^) or overweight or obese (≥24.0 kg/m^2^).

### Biochemical measurements

Fasting venous blood was collected in a 5 ml heparin anticoagulant vacuum tube and centrifuged at 20 °C and 2500 rpm. The plasma was isolated and frozen at − 20 °C, shipped in cold storage to the central laboratory at Capital Medical University in Beijing, and stored at − 80 °C until analysis.

Fasting plasma glucose, serum total cholesterol, triglycerides, high-density lipoprotein cholesterol (HDL-C) and serum hemoglobin were measured using an automatic biochemistry analyzer (7180; Hitachi, Swiss Roche Company). The plasma glucose level was measured by the glucose oxidase method. The serum total cholesterol level was measured by the cholesterol oxidase method. The serum triglyceride level was measured by the glycerol phosphate oxidase-peroxidase method. Serum hemoglobin was measured by enzyme colorimetry.

### Assessments of vitamin D status

An enzyme-linked immunosorbent assay (Immunodiagnostic Systems Limited, Bolton, UK) was used to measure plasma 25-hydroxyvitamin D [25(OH)D] levels, and the inter- and intra-assay coefficients of variation were less than 10 and 8%, respectively. Serum vitamin D levels were defined as “sufficient” (25(OH)D ≥ 30 ng/mL), “insufficient” (20 ≤ 25(OH)D < 30 ng/mL), or “deficient” (< 20 ng/mL) [16].

### Classification of MetS

ATP III recommended obesity be the primary target of intervention for metabolic syndrome. First-line therapy should be weight reduction reinforced with increased physically active. Weight loss can lower serum cholesterol and triglycerides, blood pressure and glucose, and raise HDL cholesterol, and reduce insulin resistance. We used the following three ethnicity-specific MetS criteria to compute the prevalence of MetS and study the relationship between vitamin D level and MetS [17]: (1) the Adult Treatment Panel III of the National Cholesterol Education Program (ATP III) guidelines, modified in accordance with the World Health Organization (WHO)‘s proposed WC cutoff points for Asians (modified ATP III) [18], (2) the Chinese Diabetes Society (CDS) criteria [19], and (3) the modified CDS criteria (2012) [20] (Tables 1 and 2). The modified ATP III guidelines have been widely used in the study of the relationship between vitamin D and MetS in various countries. The CDS criteria were applicable diagnostic criteria for the Chinese population based on the study of MetS in China, which was proposed by the Diabetes Society of the Chinese Medical Association in 2004. The modified criteria (2012) is a simplified “modified” explanatory model to predict MetS by differentially examining the risk of cardiometabolic syndrome in Asians. Increased WC plus one other MetS component was required for diagnosis [20].

**Table 1:** Definition of metabolic syndrome

Criteria	Modified ATP III Guidelines	Chinese Diabetes Society (CDS) Criteria	Modified Criteria (2012)
	At least 3 of the following factors listed below	3 or 4 of the following factors listed below	Waist circumference measurement (below) plus at least 1 of the following factors listed below
Fasting glucose	≥100 mg/dL or diagnosis of type 2 diabetes	≥110 mg/dL or known treatment for diabetes	≥100 mg/dL or diagnosis of type 2 diabetes
Waist circumference	≥90 cm for males and ≥ 80 cm for females	Not applied	≥90 cm for males and ≥ 80 cm for females
Blood pressure	≥130/85 mmHg or known treatment for hypertension	≥140 /90 mmHg or known treatment for hypertension	≥130/85 mmHg or known treatment for hypertension
Serum triglycerides	≥150 mg/dL or treatment for the same	triacylglycerol ≥150 mg/dL or high-density lipoprotein cholesterol < 35 mg/dL for males and < 39 mg/dL for females	≥150 mg/dL or treatment for the same
Serum high-density lipoprotein cholesterol	< 40 mg/dL for males and < 50 mg/dL for females or treatment for the same		< 40 mg/dL for males and < 50 mg/dL for females or treatment for the same
Body mass index	Not applied	≥25 kg/m^2^	Not applied

**Table 2:** Basic characteristics of the study subjects

Characteristics	25(OH)D status			***P-***value
	Sufficient(≥30 ng/ml) ***n*** = 574	Insufficiency(20-30 ng/ml) ***n*** = 890	Deficiency(< 20 ng/ml) ***n*** = 1029	
MetS[Modified ATP III(2009)] (%)	218 (39.1)	373 (42.9)	484 (48.6)	< 0.01
MetS[Modified Criteria(2012)] (%)	241 (46.8)	422 (51.6)	506 (53.0)	0.07
MetS(CDS) (%)	124 (22.5)	214 (25.4)	295 (32.5)	< 0.01
Age group(≥80 years old) (%)	291 (50.7)	502 (56.4)	766 (74.4)	< 0.01
Female(%)	174 (30.3)	419 (47.1)	703 (68.3)	< 0.01
Education(%)				
illiteracy	130 (40.8)	233 (48.6)	405 (63.0)	< 0.01
1-9 years	170 (53.3)	228 (47.6)	218 (33.9)	
≥ 10 years	19 (6.0)	18 (3.8)	20 (3.1)	
Nationality(han) (%)	432 (84.7)	698 (91.5)	858 (94.8)	< 0.01
Married(%)	352 (61.5)	434 (48.9)	332 (32.4)	< 0.01
Smoking (%)	135 (23.6)	147 (16.6)	152 (14.9)	< 0.01
Drinking (%)	147 (25.8)	150 (17.0)	139 (13.6)	< 0.01
Physically active (%)	532 (96.0)	818 (93.6)	910 (90.6)	< 0.01
Elevated WHR (%)	391 (68.1)	650 (73.1)	792 (77.0)	< 0.01
Elevated BMI (%)	190 (33.5)	298 (34.5)	292 (31.2)	< 0.01
Elevated WC (%)	263 (45.8)	468 (52.6)	557 (54.1)	< 0.01
Rural (%)	505 (96.7)	740 (95.4)	849 (92.9)	< 0.01
Season(%)				
Spring(3–5)	7 (1.3)	15 (1.8)	14 (1.5)	0.09
Summer(6–8)	1 (0.2)	0 (0)	0 (0)	
Autumn(9–11)	414 (76.0)	670 (78.3)	766 (81.5)	
Winter(12–2)	123 (22.6)	171 (20.0)	160 (17.0)	
Age(years)	80.6 ± 10.1	83.7 ± 29.1	89 ± 27.4	< 0.01
Systolic blood pressure(mmHg)	149.5 ± 22.1	148.2 ± 23.3	148.3 ± 24.6	0.54
Diastolic blood pressure(mmHg)	83.3 ± 12.6	82.3 ± 12.4	81.4 ± 12.7	0.02
Fasting plasma glucose(mmol/L)	5.4 ± 1.8	5.5 ± 1.7	6.9 ± 4.3	< 0.01
Glycosylated serum protein (μmol/L)	255.1 ± 44.1	259.7 ± 37.8	246.9 ± 61.6	< 0.01
BMI(kg/m^2^)	22.5 ± 3.5	22.7 ± 3.8	22.5 ± 7.3	0.71
Triglyceride(mmol/L)	1.3 ± 0.7	1.4 ± 0.9	1.7 ± 1.4	< 0.01
HDL cholesterol(mmol/L)	1.5 ± 0.4	1.4 ± 0.4	1.4 ± 0.4	0.05
Total cholesterol(mmol/L)	4.7 ± 0.9	4.8 ± 1	4.8 ± 1	0.27
weight (Kg)	56.4 ± 11.7	54.8 ± 12.6	51.1 ± 12.6	< 0.01
Height(cm)	157.7 ± 10	154.9 ± 10.3	150.7 ± 11	< 0.01
Waist circumference(cm)	85.4 ± 9.9	85.7 ± 10.6	83.9 ± 11.2	< 0.01
WHR	0.92 ± 0.08	0.92 ± 0.08	0.92 ± 0.07	0.93

Notes: Data are mean ± SE

*25(OH)D* 25 hydroxy vitamin D, *MetS(CDS)* metabolic syndrome based on CDS; MetS[Modified ATP III (2009)], MetS(CDS), MetS [Modified ATP III (2009)], metabolic syndrome based on modified ATP III (2009); MetS[Modified Criteria (2012)], metabolic syndrome based on modified criteria (2012);*WHR* waist-hip ratio, *BMI* body mass index, *WC* waist circumference(cm), *HDL* high density lipoprotein

### Statistical analysis

Continuous variables are presented as means ± SDs and were analyzed with one-way ANOVA. Categorical variables are presented as percentages and were analyzed with the chi-square test. Logarithmic transformation was applied when the continuous variables did not conform to a normal distribution.

The variables were categorized as follows: age was stratified into 10-year periods, and waist-hip ratio was classified as < 0.9 or ≥ 0.9 for males and < 0.85 or ≥ 0.85 for females [21]. BMI was classified as < 24.0 or ≥ 24.0 kg/m^2^. Multivariate logistic regression analyses were performed to investigate the association between vitamin D status and MetS. Adjustments were made for age, sex, education level (0, 1–9 or ≥ 10 years), marital status (married or single), race (Han or other ethnic minority), BMI (< 24.0 kg/m^2^ or ≥ 24 .0 kg/m^2^), current smoking (yes or no), current alcohol consumption (yes or no), and regular physically active (yes or no). We did not consider the blood sample collection season because almost all of our samples were collected from September to November, which is autumn in China. Two models were applied for each outcome: the basic model (adjusted for age and sex) and model 2 (basic model plus marital status and health-related lifestyle factors such as smoking, alcohol consumption, physically active and BMI). We mainly explored the relationship between vitamin D and MetS according to the modified ATP III guidelines for MetS to allow for comparisons with other similar studies. We also explored the relationship between vitamin D and MetS based on two other MetS diagnostic criteria. The associations between vitamin D status and individual components of MetS were investigated with the full model, and the associations between vitamin D status and individual components of MetS were further tested with a multivariate model after further adjustment for the remaining components of MetS.

To evaluate the robustness of our results, we conducted sensitivity analyses by individually excluding some participants (if they suffered from multiple diseases, had inadequate mobility, or were under 80 years old) from the model. All statistical tests were two-tailed, with an alpha = 0.05 level of significance, and were performed using SAS 9.4 software (SAS Institute Inc., Cary, NC).

## Results

### Baseline characteristics of the study population

**Fig. 1:**
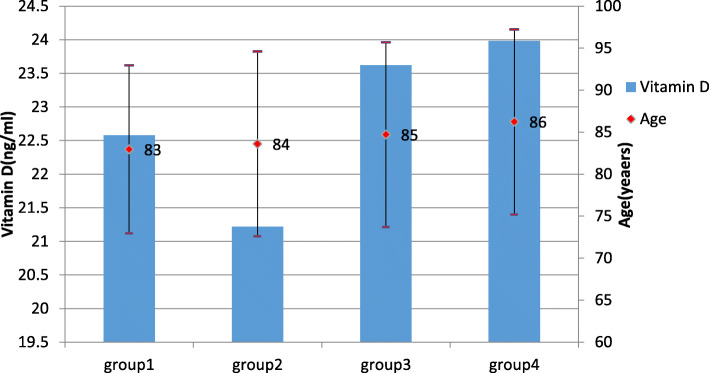
Ages and vitamin D levels of subjects with MetS based on different diagnostic criterias. Notes:group1: Non-metabolic syndrome population;group2: MetS based on modified ATP III while without MetS based on modified criteria (2012); group3: MetS based on modified criteria (2012) while without MetS based on modified ATP III;group4:MetS based on both modified ATP III and Modified Criteria (2012)

The present study population was composed of 2493 participants, with an average age of 85 years, and included 1559 elderly people over the age of 80 years old, accounting for 62.5% of all the subjects, and consisted of 1197 males and 1296 females. The proportions of people with vitamin D insufficiency (20 ≤ 25(OH)D < 30 ng/mL) and vitamin D deficiency (25(OH)D < 20 ng/ml) were 35.7 and 41.3%, respectively. Their average age was 85.7 years, which was older than that of the vitamin D-sufficient group (*p* < 0.01). The average age of MetS according to ATP III,2012 and CDS was 85, 85 and 82 years old respectively. The level of vitamin D in elderly individuals over 100 years old was 17.95 ng/ml. The level of vitamin D of MetS according to ATP III,2012 and CDS was 22.3,22.9 and 21.7 ng/ml respectively. The other characteristics of the total study population are shown in Tables 1 and 2. The vitamin D level of people with MetS based on the modified ATP III criteria but without MetS based on the modified criteria (2012) was 21.2 ng/ml, which was significantly lower than that of individuals with MetS based on the modified criteria (2012) but without MetS based on the modified ATP III criteria (23.6 ng/ml). The ages of these two groups were 84 and 85 years, respectively. The other characteristics of different group classified based on a combination of different diagnostic criteria are shown in Fig. 1.

There was no significant difference in systolic blood pressure or HDL-C among the vitamin D-sufficient group, the vitamin D-insufficient group and the vitamin D-deficient group (*p* > 0.05). The weight, height, triglyceride levels, fasting blood sugar and WC were significantly different among the three groups (*p* < 0.05). Moreover, there were significant differences in the waist-hip ratio among the three groups (*p* < 0.05). The percentage of people with an appropriate waist-hip ratio was higher in the vitamin D-sufficient group than in the other two groups. The prevalence of MetS was lowest in the vitamin D-sufficient group and highest in the vitamin D-deficient group regardless of the criteria used for the diagnosis of MetS. The prevalence of MetS according to ATP III,2012 and CDS was 44.4%,51.1 and 27.5% respectively. The difference in the prevalence of MetS based on CDS,ATP III and 2012 was statistically significant between each group (*p* < 0.01).

### The relationship between vitamin D status and MetS

In the analysis of the basic model (model 1), with adjustments for only age and sex, each 10 ng/ml increase in serum 25(OH)D was significantly associated with a decreased prevalence of MetS according to the modified ATP III criteria (OR: 0.92, 95% CI: 0.84, 1.00). After adjusting for other potential confounders, including nationality, education level, marital status, smoking, alcohol consumption, regular physically active and BMI (model 2), the results were still statistically significant (OR: 0.79, 95% CI: 0.69, 0.90). The vitamin D-sufficient group had a lower risk of MetS the vitamin D-deficient group based on model 2 (OR: 0.63, 95% CI: 0.52, 0.92). We applied the same analysis of MetS according to the CDS criteria and the modified criteria (2012). A similar significant relationship between vitamin D levels and MetS based on the CDS criteria was found. No statistically significant association was found between vitamin D levels and MetS based on the modified criteria (2012) (see Table 3 for details).

**Table 3:** Multiple logistic regression and subgroup analysis for the association of metabolic syndrome based on different diagnostic criterias with vitamin D status

Variables	25(OH)D[OR (95%CI)]			
	Continuous	< 20 ng/ml	20—30 ng/ml	≥30 ng/ml
MetS [modified ATP III (2009)]				
Model1	0.92 (0.84to1.00)	reference	0.88 (0.73to1.07)	0.83 (0.66to1.04)
Model2	0.79 (0.69to0.90)	reference	0.70 (0.52to0.92)	0.63 (0.45to0.88)
MetS[Modified Criteria(2012)]				
Model1	1.08 (0.99to1.18)	reference	1.13 (0.92to1.38)	1.07 (0.85to1.35)
Model2	1.08 (0.94to1.24)	reference	0.94 (0.68to1.30)	1.06 (0.73to1.55)
Mets (CDS)				
Model1	0.70 (0.63to0.78)	reference	0.50 (0.36to0.79)	0.54 (0.41to0.70)
Model2	0.63 (0.54to0.74)	reference	0.71 (0.60to0.69)	0.46 (0.31to0.69)
Subgroup of normal WC^a^				
Model1	0.71 (0.60to0.85)	reference	0.53 (0.37to0.77)	0.53 (0.35to0.81)
Model2	0.55 (0.43to0.71)	reference	0.49 (0.29to0.84)	0.28 (0.14to0.55)
Subgroup of elevated WC^a^				
Model1	0.93 (0.82to1.06)	reference	0.94 (0.71to1.24)	0.90 (0.65to1.25)
Model2	0.84 (0.69to1.01)	reference	0.79 (0.53to1.18)	0.81 (0.50to1.32)

Notes: *25(OH)D* 25 hydroxy vitamin D, MetS[Modified ATP III (2009)], Metabolic syndrome based on modified ATP III (2009);MetS[Modified Criteria (2012)], Metabolic syndrome based on Modified Criteria (2012); *WC* waist circumference(cm)

Model1,adjusted for age,sex

Model2,adjusted for age, sex, race, education, marrage, physically active, smoking, drinking, BMI

^a^Metabolic syndrome is diagnosied with modified ATP III (2009)

### The relationships between vitamin D and the individual components of MetS

We also analyzed the relationships of each MetS component and waist-hip ratio with vitamin D. Each 10 ng/ml increase in serum vitamin D was significantly associated with a decreased risk of elevated triglycerides (OR: 0.60, 95% CI: 0.51, 0.71), reduced HDL-C (OR: 0.67, 95% CI: 0.58,0.78) and elevated glucose (OR: 0.88, 95% CI: 0.78, 0.99) based on model 2. The vitamin D-sufficient group had a lower risk of elevated triglycerides (OR: 0.38, 95% CI: 0.25,0.56) and reduced HDL-C (OR: 0.47,95% CI: 0.32, 0.69) than the vitamin D-deficient group based on model 2(see Table 4 for details). After further adjustment for other components of MetS, we still found significant associations between each 10 ng/ml increase in serum 25(OH)D and elevated triglycerides, reduced HDL-C and elevated glucose. The vitamin D-sufficient group had a lower risk of elevated triglycerides (OR: 0.46, 95% CI: 0.30, 0.71) and reduced HDL-C (OR: 0.64, 95% CI: 0.42, 0.97) than the vitamin D-deficient group after further adjustment for other components of MetS (see Table 5 for details).

**Table 4:** Multiple logistic regression for the association of individual Components of metabolic syndrome with vitamin D status

Variables	25(OH)D[OR (95%CI)]			
	Continuous	< 20 ng/ml	20-30 ng/ml	≥30 ng/ml
Elevated triglycerides	0.60 (0.51to0.71)	reference	0.54 (0.40to0.74)	0.38 (0.25to0.56)
Reduced HDL cholesterol	0.67 (0.58to0.78)	reference	0.66 (0.50to0.89)	0.47 (0.32to0.69)
Elevated glucose	0.88 (0.78to0.99)	reference	0.86 (0.66to1.12)	0.80 (0.59to1.10)
Elevated WC	1.08 (0.95to1.24)	reference	1.00 (0.73to1.37)	1.04 (0.72to1.51)
Elevated BP	1.19 (1.02to1.39)	reference	1.15 (0.82to1.62)	1.34 (0.89to2.03)

Notes: Multivariate model is adjust for age, sex, race, education, marrage, physically active, smoking, drinking, BMI

*25(OH)D* 25 hydroxy vitamin D, *HDL* high density lipoprotein. *WC* waist circumference(cm), *BP* blood pressure

**Table 5:** Multiple logistic regression for the association of individual Components of metabolic syndrome with vitamin D status after adjustment of the other MetS components

Variables	25(OH)D[OR (95%CI)]			
	Continuous	< 20 ng/ml	20—30 ng/ml	≥30 ng/ml
Elevated triglycerides	0.70 (0.59to0.82)	reference	0.59 (0.42to0.83)	0.46 (0.30to0.71)
Reduced HDL cholesterol	0.74 (0.63to0.87)	reference	0.81 (0.59to1.13)	0.64 (0.42to0.97)
Elevated glucose	0.84 (0.75to0.95)	reference	0.86 (0.65to1.14)	0.73 (0.53to1.01)
Elevated waist circumference	1.09 (0.94to1.25)	reference	0.96 (0.70to1.33)	1.05 (0.72to1.54)
Elevated blood pressure	1.26 (1.07to1.49)	reference	1.23 (0.87to1.75)	1.49 (0.97to2.29)

Notes: Multivariate model is adjust for age, sex, race, education, marrage, physically active, smoking, drinking, BMI, plus additionally adjusted for the rest of the individual components of the MetS

*25(OH)D* 25 hydroxy vitamin D, *HDL* high density lipoprotein. *WC* waist circumference(cm), *BP* blood pressure

### Sensitivity analysis

Table 6 shows the results of the sensitivity analyses. When individually excluding participants (if they suffered from multiple diseases, had inadequate mobility, or were younger than 80 years old) from the model, the estimates of the ORs for vitamin D and MetS remained statistically significant and were very similar to the estimates in the main analyses.

**Table 6:** Sensitivity analysis for the association of metabolic syndrome based on different diagnostic criterias with vitamin D status

Variables	25(OH)D[OR (95%CI)]			
	Continuous	< 20 ng/ml	20—30 ng/ml	≥30 ng/ml
Exclude people with combined diseases				
MetS [modified ATP III]	0.7 (0.58to0.86)	reference	0.58 (0.38to0.88)	0.53 (0.32to0.88)
MetS[Modified Criteria(2012)]	1.14 (0.94to1.38)	reference	1.00 (0.64to1.57)	1.07 (0.64to1.79)
Mets (CDS)	0.48 (0.36to0.65)	reference	0.33 (0.18to0.60)	0.37 (0.19to0.73)
Exclude people with inadequate mobility				
MetS [modified ATP III]	0.73 (0.62to0.86)	reference	0.62 (0.45to0.87)	0.54 (0.36to0.81)
MetS[Modified Criteria(2012)]	0.73 (0.62to1.27)	reference	0.62 (0.45to1.23)	0.54 (0.36to1.57)
Mets (CDS)	0.62 (0.51to0.76)	reference	0.5 (0.34to0.73)	0.48 (0.30to0.76)
Exclude people under 80 years old				
MetS [modified ATP III]	0.86 (0.74to1.02)	reference	0.78 (0.55to1.11)	0.76 (0.48to1.18)
MetS[Modified Criteria(2012)]	1.06 (0.89to1.26)	reference	0.90 (0.60to1.35)	1.00 (0.60to1.65)
Mets (CDS)	0.68 (0.55to0.84)	reference	0.62 (0.40to0.96)	0.54 (0.31to0.96)

Notes: Multivariate model is adjust for age, sex,race,education,marrage, physically active,smoking,drinking,BMI

## Discussion

We found that vitamin D deficiency was significantly associated with an increased risk of MetS based on the modified ATP III criteria and CDS criteria in elderly individuals. However, we did not find any statistical association between vitamin D deficiency and MetS based on the modified criteria (2012). We also found that vitamin D deficiency was inversely associated with the prevalence of elevated serum triglycerides and reduced HDL-C. No significant association was found between vitamin D deficiency and other components of MetS.

The majority of previous studies on the relationship of vitamin D with MetS among Chinese populations focused on middle-aged and elderly people, with an average age younger than 60 years [12, 22]. Some similar studies in other countries included those conducted in the United States [10] and the Netherlands [23], with average participant ages of 73.5 years and 71.2 years, respectively. The average age of our subjects was 85.2 years old. MetS is associated with a variety of adverse health outcomes, including type 2 diabetes, cardiovascular disease, cancer and so on, which affect life expectancy in elderly individuals [24–26]. Among the elderly population, especially longaevous individuals, there are great differences in metabolic capacity, living habits, such as outdoor exercise time, and activity ability between elderly individuals and those younger than 60 years [10]. Moreover, with increasing age among the elderly, the ability to absorb and metabolize vitamin D decreases and body fat gradually accumulates, increasing the distributed volume and decreasing the biological activity of fat-soluble vitamin D in obese people [27]. Therefore, there are some characteristics of the relationship between vitamin D and MetS among the elderly and longevity groups that have been studied for the first time.

We also found that vitamin D deficiency and insufficiency were very common in the oldest-old adults; with increased age, serum vitamin D levels decrease. The serum vitamin D levels in elderly individuals over 100 years old was much lower than that reported among Chinese elderly individuals in Shanghai by Wei [12] in 2014, even though our sample collection occurred mainly concentrated in autumn when serum vitamin D levels are relatively high. This figure was slightly higher than 16.06 ng/ml in Chinese elderly individuals, as reported by Ling [9] and was similar to the level in those at an average age of 79 years in Taiwan [28]. The prevalence of vitamin D deficiency and insufficiency was 41.3 and 35.7%, respectively, which was lower than previously reported results [9, 10, 23] and was especially lower than the results of the study conducted in Lanzhou, China [6]. This deviation may be derived in part from the fact that some of the subjects came from southern provinces in China where serum vitamin D levels are higher than those in the north. In addition, some studies [29] have shown that serum vitamin D was associated with total mortality in the general population. There may be a survivor bias for people over 100 years old who were included in our study.

The most accepted criteria for defining MetS was first proposed by the WHO in 1999 [30]. The major academic groups in the world, including the American Association of Clinical Endocrinologists (AACE) [31], ATP III [18], the Third Report of the National Cholesterol Education Program Expert Panel on Detection, the European Group for the Study of Insulin Resistance (EGIR) [32], and the International Diabetes Federation (IDF) [33], have proposed many different diagnostic definitions of MetS. Whether abdominal obesity should be used as a necessary diagnostic component has always been a topic of debate. The criteria change with varying study duration, demographic area, age and disease prediction ability [34, 35]. We studied the relationship of vitamin D with MetS based on different criteria simultaneously and further explored the causes of the differences.

We selected the latest three MetS criteria, among which WC or BMI criteria were modified in accordance with the WHO’s proposed cutoff points for Asians. The prevalence of MetS according to CDS, ATP III and 2012 were diffierent. We found similar statistical correlations between vitamin D levels and MetS based on the CDS and modified ATP III criteria [6, 8, 10, 11, 13, 14]. There were no significant correlations between vitamin D and MetS based on the modified criteria (2012) or the IDF (results not shown). The results remained the same whether various confounding factors were adjusted for or vitamin D levels were analyzed as continuous variables. A significant correlation between vitamin D and MetS was usually based on the ATP III or modified ATP III criteria in other similar studies [9, 10, 22]. Different diagnostic criteria may focus on different pathological mechanisms. The CDS and modified ATP III criteria include obesity as an equally important component as other components in the diagnosis, assuming that the pathological mechanism of MetS extends from insulin resistance to a syndrome consisting of obesity and adipose tissue metabolic disorders [36]. However, the modified criteria (2012) and the IDF regard WC as a necessary prerequisite, and the remaining components are equally important, which means that the criteria emphasize a pathological mechanism centered on central obesity and is applicable to obesity to groups with disorders of glucose and lipid metabolism. The ability to predict possible adverse outcomes in the future is an important factor that must be considered when proposing diagnostic criteria for MetS. Some previous studies have shown that the ability to predict the risk of cardiovascular disease and type 2 diabetes mellitus associated with MetS diagnosed by the modified ATP III criteria is better than MetS diagnosed considering abdominal obesity a necessary diagnostic criterion. The ATP III criteria clearly identifies the burden of coronary heart disease associated with MetS [37].

We found that the vitamin D level of people with MetS based on the modified ATP III criteria but without MetS based on the modified criteria (2012) was 21.22 ng/ml, which was significantly lower than that of individuals with MetS based on the modified criteria (2012) but without MetS based on the modified ATP III criteria (23.63 ng/ml), so the clustering characteristics of the MetS risk components related to vitamin D level changed in the elderly population. The component analysis revealed that rather than central obesity, vitamin D affected MetS mainly by affecting blood lipid metabolism. More mechanisms and prospective studies are needed to determine which diagnostic criteria are appropriate for different age groups when studying the relationship between vitamin D and MetS.

Some previous studies have shown that there are sex differences in the relationship between vitamin D and MetS [37, 38]. Subgroup analyses were carried out for gender, WC (increased or not) and BMI (increased or not) groups. The results showed that the ORs were similar between the different gender groups and BMI groups (results not shown). This finding may be related to the fact that all the women involved in our study were postmenopausal. A significant statistical correlation was found among elderly people with a normal WC, while no significant statistical correlation was found among elderly people with an increased WC. Vitamin D is a fat-soluble vitamin and is involved in fat metabolism. The lipid components played a very important role in the development of metabolic syndrome. This indicates that difference in fat distribution in the body may affect vitamin D metabolism and the relationship between metabolic syndrome and vitamin D. Some studies [39, 40] have shown that vitamin D deficiency is closely related to body fat content and body fat distribution.

Many studies have found that vitamin D levels are significantly associated with blood lipid levels, and we found that vitamin D levels exhibited a significant correlation with triglycerides and HDL-C. Similarly, Sheena [8] and Vitezova [23] also found that vitamin D levels were negatively correlated with triacylglycerol and positively correlated with HDL-C. First, vitamin D plays a very important role in the formation of HDL-C molecules [41–43]. Second, active 25(OH)D metabolites can inhibit low-density lipoprotein cholesterol (LDL-C) deposition by promoting the differentiation of monocytes or macrophages and reducing the release of proinflammatory cytokines. Third, vitamin D can regulate the influx of calcium ions, which leads to a decrease in lipase activity and inhibits lipolysis. Fourth, vitamin D can directly act on the vitamin D receptor of pre-Q adipocytes, regulate the differentiation and metabolism of adipocytes, and stimulate the synthesis and secretion of lipoprotein lipase [44]. Finally, vitamin D can also reduce the synthesis and secretion of parathyroid hormone (PTH), enhance lipolysis activity and improve abnormal lipid metabolism [45–47].

### Strengths

The study included the oldest population, including elderly people mostly over 80 years of age, to study the comprehensive relationships between vitamin D and MetS. We studied the relationship between vitamin D and MetS based on three MetS diagnostic criteria.

### Limitations

There are several limitations to the study. First, the results are based data obtained with a cross-sectional study design. We revealed a link between only vitamin D and MetS but could not ascertain the causality of the result. Second, almost all of our biological samples were collected during the same season, so we could not assess the impact of seasonal factors on outcomes. Third, the relationship between vitamin D and metabolic function may be different in different age groups, so our findings are applicable to only the elderly population. In addition, a significant relationship between vitamin D and triglycerides as well as HDL-C was found, but no further measurement of PTH levels was performed. Finally, we adjusted for potential confounders, but we could not estimate the effects of other unmeasured potential confounding factors.

## Conclusions

A significant correlation between vitamin D and MetS in the elderly population in China was found and was significant in those with concealed obesity. Additional attention needs to be paid to the level of vitamin D in the population with normal WC in the context of the prevention and treatment of MetS. Appropriate diagnostic criteria for MetS need to be chosen based on the characteristics of the study population to explore the relationship between vitamin D and MetS in future studies.

## Data Availability

This study was based on the datasets from the Chinese Longitudinal Healthy Longevity Survey (CLHLS) in longevity areas. The CLHLS datasets are publicly available at the National Archive of Computerized Data on Ageing (NACDA),University of Michigan(https://www.icpsr.umich.edu/icpsrweb/NACDA/series/487). Researchers can obtain these data after submitting a data use agreement to CLHLS team.

## Abbreviations

MetS: Metabolic syndrome
BMI: Body mass index
CLHLS: Chinese Longitudinal Healthy Longevity Survey (CLHLS)
WC: Waist circumference
HDL-C: High-density lipoprotein cholesterol
WHO: The World Helath Orgnization
CDS: The Chinese Diabetes Society
EGIR: The European Group for the study of Insulin
IDF: The International Diabetes Federation
CVD: Cardiovascular disease
AACE IRS: American Association of Clinical Endocrinologists
ATP III: The Adult Treatment Panel III of the National Cholesterol Education Program guidelines
PTH: Parathyroid hormone
